# An extremely rare case of nivolumab‐associated macroscopic duodenitis with spontaneous regression

**DOI:** 10.1002/rcr2.582

**Published:** 2020-05-11

**Authors:** Takatora Akizawa, Takeshi Saraya, Hiroki Takakura, Masachika Fujiwara, Haruyuki Ishii, Hajime Takizawa

**Affiliations:** ^1^ Department of Respiratory Medicine Kyorin University School of Medicine Tokyo Japan; ^2^ Department of Pathology Kyorin University School of Medicine Tokyo Japan

**Keywords:** Duodenitis, immune‐related adverse events, nivolumab, spontaneous regression

## Abstract

An 82‐year‐old man was presented to our hospital due to epigastric and right hypochondrial pain 17 weeks after the initiation of intravenous treatment with nivolumab for recurrent lung adenocarcinoma as multiple lung and sternal metastases. Urgent gastroscopy revealed macroscopic duodenitis such as severe erythema, oedema, black‐coloured erosions, and ulcers located throughout the second portion of the duodenum, which was confirmed by abdominal computed tomography as circumferential thickening of the duodenal wall. Those lesions were pathologically considered as non‐specific inflammation and spontaneously disappeared within a month, suggesting nivolumab‐induced immune‐related adverse events.

## Graphical abstract

Immune‐related adverse events usually occur in the colon; however, the duodenum could be affected by the immune checkpoint inhibitors. This is an extremely rare phenomenon that is characterized by pathologically non‐specific inflammation.

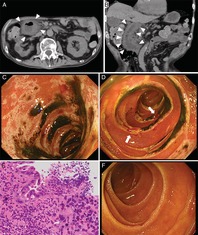

## Introduction

Immune‐related adverse events (irAE) usually occur in the colon; however, the duodenum could be affected by the immune checkpoint inhibitors. This is an extremely rare phenomenon that is characterized by pathologically non‐specific inflammation.

## Case Report

An 82‐year‐old man was admitted to our hospital with complaints of abdominal pain and loss of appetite for the past three weeks. Four years prior, he was diagnosed with pulmonary adenocarcinoma (cT2aN2M0 stage IIIA), and a right upper lobectomy was performed. The carcinoma cells were epidermal growth factor receptor wild‐type cells and negative for both anaplastic lymphoma kinase and programmed death‐ligand 1 (PD‐L1). Two years later, multiple lung and sternal metastases recurred and were treated orally with the anti‐cancer drug S‐1; however, cancer progressed, and intravenous nivolumab treatment was started four months prior to this admission. The patient was a former smoker with a 60 pack‐year history.

**Figure 1:**
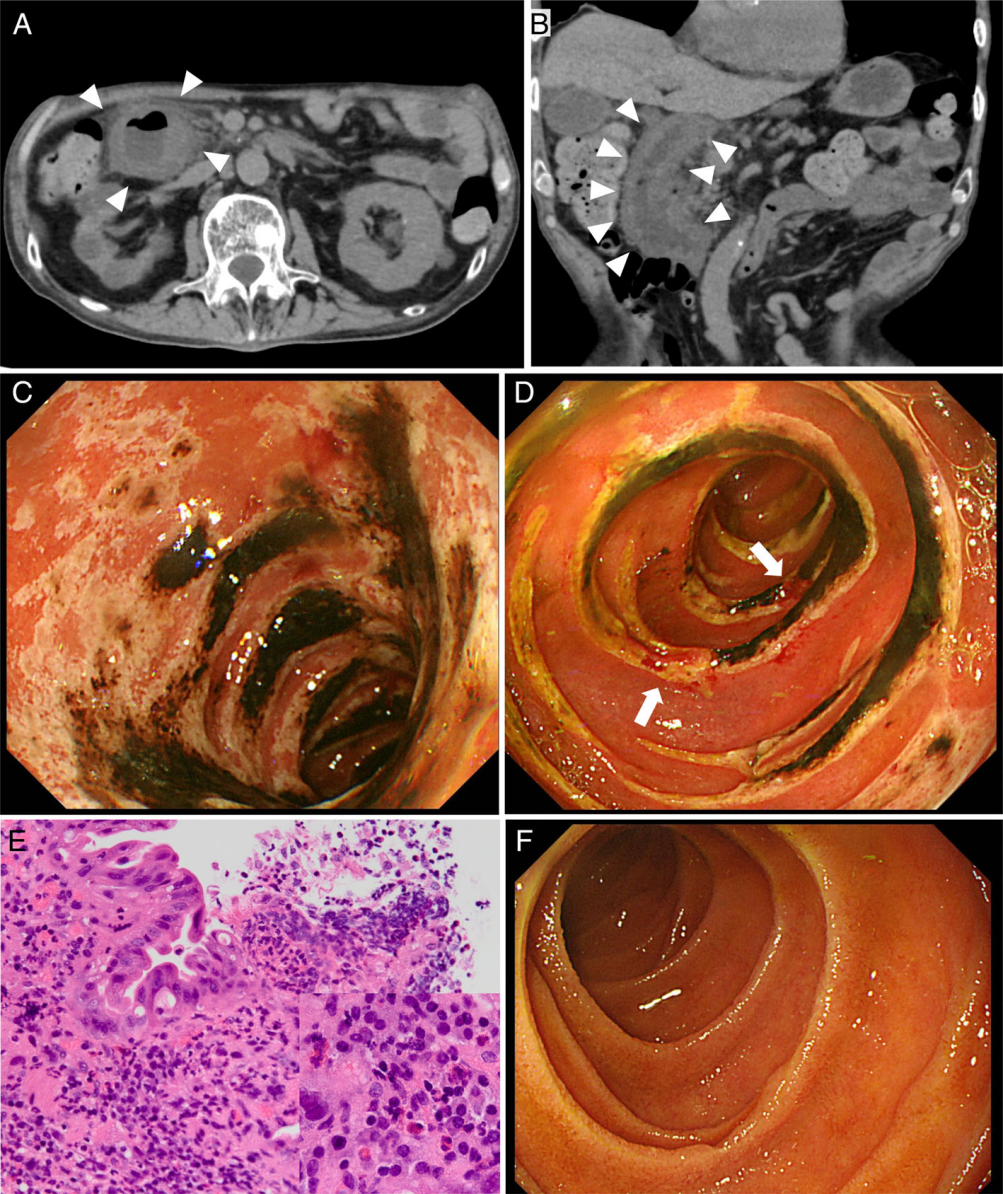
(A) Computed tomography image shows an axial view of the thickened wall of the second portion of the duodenum (arrowheads). (B) Computed tomography image shows a coronal view of the thickened wall of the second portion of the duodenum (arrowheads). (C) Gastroscopic image showing severe erythema, oedema, and black‐coloured erosions in the second portion of the duodenum. (D) Gastroscopic image showing multiple ulcers (arrows) in the second portion of the duodenum. (E) Microscope image (200×) showing surface epithelium desquamation with abundant neutrophilic and lymphocytic infiltration and focal accumulation of eosinophils (inset). (F) Gastroscopic image of the complete resolution of previous findings.

On admission, vital signs were normal, and a physical examination revealed only epigastric and right hypochondrial pain without rebound tenderness. Serum laboratory data showed moderate to marked elevation in white blood cells (15,000/μL), C‐reactive protein (12.7 mg/dL), lactase dehydrogenase (798 IU/L), blood urea nitrogen (57.0 mg/dL), and creatinine (1.44 mg/dL). Abdominal computed tomography revealed circumferential thickening in the second portion of the duodenal wall in both axial (Fig. 1A, arrowheads) and coronal views (Fig. 1B, arrowheads). Urgent gastroscopy revealed severe erythema, oedema, and black‐coloured erosions (Fig. 1C) with scattered ulcers (Fig. 1D, arrows) located throughout the second portion of the duodenum but limited from the bulb to the beginning of the second portion. The haematoxylin–eosin stained biopsy specimens obtained from the second portion of the duodenum were viewed at 200× (Fig. 1E) and showed surface epithelium desquamation with abundant neutrophilic and lymphocytic infiltration and focal accumulation of eosinophils (Fig. 1E, inset), indicating non‐specific inflammation.

On the basis of these findings, the patient was diagnosed with nivolumab‐induced irAEs limited to the second portion of the duodenum. After treatment comprising three days of fasting, fluid therapy, and proton pump inhibitor administration, his symptoms improved gradually, leading to a complete resolution of the gastroscopic findings (Fig. 1F). Three months after the onset of irAE, the multiple lung and sternal metastases showed stable disease; however, his general condition gradually deteriorated, thereby he was transferred to another hospital for palliative care.

## Discussion

The first case of severe macroscopic duodenitis with spontaneous resolution (diagnosed as irAE) was confirmed by radiological, pathological, and endoscopic findings 17 weeks after nivolumab treatment.

Approximately 10% of all patients receiving nivolumab are diagnosed with irAE within 16 weeks [1]. It most commonly affects the skin, gastrointestinal, endocrine, and pulmonary systems and appears as colitis when gastrointestinal lesions appear on the descending colon. The present case developed irAE‐duodenitis 17 weeks after nivolumab treatment; however, the exact timeframe in which it occurred is unknown. To the best of our knowledge, there are limited reports on irAE‐duodenitis so far; however, previous reports state that upper gastrointestinal disorders occurred in less than 1% of all melanoma patients who received nivolumab [2, 3].

Clinical symptoms in irAE‐colitis patients include diarrhoea, abdominal pain, and haematochezia, whereas clinical symptoms in irAE‐duodenitis patients include loss of appetite, epigastric pain, dark stool, and diarrhoea. The patient in our report had only two of the four symptoms mentioned above.

Gonzalez et al. reported that the most common finding in irAE‐duodenitis patients who received PD‐1/PD‐L1 was expanding eosinophil infiltration in the lamina propria [4]. Histological findings of our case were partially consistent with their report, but the inflammatory response was rather non‐specific. The irAE guidelines stated that corticosteroids should be used for irAE‐colitis and PD‐1/PD‐L1 inhibitors or anti‐cytotoxic T lymphocyte‐associated antigen‐4 antibodies should be discontinued in patients with grade II or higher toxicities [5]. No treatment options for irAE‐duodenitis were noted. Our case clearly demonstrated that severe macroscopic irAE‐duodenitis completely resolved without administering steroids, which indicated that irAE was a transient, upgraded immune response related to the use of nivolumab. Hence, physicians should be aware of the organs associated with irAE according to the immune checkpoint inhibitors.

### Disclosure Statement

Appropriate written informed consent was obtained for publication of this case report and accompanying images.
